# Genome-scale analysis of syngas fermenting acetogenic bacteria reveals the translational regulation for its autotrophic growth

**DOI:** 10.1186/s12864-018-5238-0

**Published:** 2018-11-23

**Authors:** Yoseb Song, Jongoh Shin, Sangrak Jin, Jung-Kul Lee, Dong Rip Kim, Sun Chang Kim, Suhyung Cho, Byung-Kwan Cho

**Affiliations:** 1 Department of Biological Sciences, Korea Advanced Institute of Science and Technology, Daejeon, 34141 Republic of Korea; 2 Department of Chemical Engineering, Konkuk University, Seoul, 05029 Republic of Korea; 3 Department of Mechanical Engineering, Hanyang University, Seoul, 04763 Republic of Korea; 4 KAIST Institute for the BioCentury, Korea Advanced Institute of Science and Technology, Daejeon, 34141 Republic of Korea; 5 Intelligent Synthetic Biology Center, Daejeon, 34141 Republic of Korea

**Keywords:** Acetogenic bacteria, *Eubacterium limosum*, Gas fermentation, Wood-Ljungdahl pathway, Translation efficiency

## Abstract

**Background:**

Acetogenic bacteria constitute promising biocatalysts for the conversion of CO_2_/H_2_ or synthesis gas (H_2_/CO/CO_2_) into biofuels and value-added biochemicals. These microorganisms are naturally capable of autotrophic growth via unique acetogenesis metabolism. Despite their biosynthetic potential for commercial applications, a systemic understanding of the transcriptional and translational regulation of the acetogenesis metabolism remains unclear.

**Results:**

By integrating genome-scale transcriptomic and translatomic data, we explored the regulatory logic of the acetogenesis to convert CO_2_ into biomass and metabolites in *Eubacterium limosum*. The results indicate that majority of genes associated with autotrophic growth including the Wood-Ljungdahl pathway, the reduction of electron carriers, the energy conservation system, and gluconeogenesis were transcriptionally upregulated. The translation efficiency of genes in cellular respiration and electron bifurcation was also highly enhanced. In contrast, the transcriptionally abundant genes involved in the carbonyl branch of the Wood-Ljungdahl pathway, as well as the ion-translocating complex and ATP synthase complex in the energy conservation system, showed decreased translation efficiency. The translation efficiencies of genes were regulated by 5′UTR secondary structure under the autotrophic growth condition.

**Conclusions:**

The results illustrated that the acetogenic bacteria reallocate protein synthesis, focusing more on the translation of genes for the generation of reduced electron carriers via electron bifurcation, rather than on those for carbon metabolism under autotrophic growth.

**Electronic supplementary material:**

The online version of this article (10.1186/s12864-018-5238-0) contains supplementary material, which is available to authorized users.

## Background

Acetogenic bacteria are naturally capable of metabolizing industrial waste gas, such as carbon monoxide (CO) and carbon dioxide (CO_2_), which are also known as components of syngas, for autotrophic growth to form biomass and to produce various metabolites, mainly acetyl-CoA, via the Wood-Ljungdahl pathway (WLP) [1, 2]. This unique capability offers a considerable potential for the sustainable production of biofuels and commodity chemicals from the abundant carbon sources. Along with several recent reports providing genomic information of these phylogenetically diverse bacteria, many biochemical studies have elucidated the molecular mechanisms associated with carbon assimilation and energy conservation pathways [3–5]. In particular, these studies revealed the existence of distinctive arrangements of WLP gene clusters encoding the carbonyl and methyl branches in acetogenic bacterial genomes. For example, *Clostridium* species carry a single gene cluster of WLP, whereas *Acetobacterium woodii* and *Eubacterium limosum* contain two sets of gene clusters that respectively consist of the methyl and the carbonyl branch associated genes [5–8]. Furthermore, different mechanisms of energy conservation between acetogenic bacteria have been proposed and partially elucidated [4–6].

Under autotrophic growth conditions (CO_2_/H_2_), acetogenic bacteria reduce two molecules of CO_2_ to acetyl-CoA using WLP, where the required electrons are derived from the oxidation of molecular hydrogen. Although the majority of genes in the methyl and carbonyl branches are well conserved in all acetogenic bacteria, two genes encoding formate dehydrogenase (FDH) and methylene-tetrahydrofolate reductase (MTHFR) are less conserved owing to the different electron-transferring mechanisms among acetogenic bacteria [9]. Acetyl-CoA resulting from the WLP is converted to acetate with the release of ATP by a substrate level phosphorylation catalyzed by a phosphotransacetylase (PTA) and an acetate kinase (ACK). As ATP is required for the formation of formyl-THF from formate in the methyl branch, the net yield of ATP generation is zero. Thus, the WLP can be regarded as an electron sink for the reduction of CO_2_ and is tightly coupled with energy conservation systems in all acetogenic bacteria. To date, two energy conservation systems have been found to generate an ion motive gradient for ATP supply (i.e., the ferredoxin (Fd):NAD^+^ oxidoreductase (Rnf) complex and the Fd:H^+^ oxidoreductase (Ech) complex). The ion used for establishing a gradient can be either Na^+^ or H^+^. Then, a membrane-bound ATP synthase complex utilizes the established ion gradient to generate ATP. In all molecular mechanisms associated with CO_2_ reduction and energy conservation, a reduced form of Fd serves as the ultimate form of electron donor in all acetogenic bacteria examined thus far. Although a large energetic barrier exists in an association with electron flow from hydrogen to Fd, acetogenic bacteria overcome the barrier using a unique flavin-based electron bifurcation [10, 11]. This system oxidizes one electron donor and transfers the electrons simultaneously to two different electron acceptors. For example, the electron-bifurcating hydrogenase in acetogenic bacteria oxidizes molecular hydrogen and catalyzes the exergonic reduction of NAD, which drives the endergonic reduction of Fd [12]. This reduced form of Fd is then used as an electron donor for an FDH and an ion-translocating membrane protein complex in all acetogenic bacteria. Notably, this understanding of the molecular details associated with acetogenesis provides the basis for engineering acetogenic bacteria to expand their capability to produce value-added biofuels and biochemicals.

Clarification of differential gene expression at the levels of transcription and translation provides important information for the genetic modification of acetogenic bacteria to increase the efficiency of inorganic carbon fixation and subsequent product formation. Toward this end, recent advances of next-generation sequencing allow expediting the analysis of less-well studied microbe gene expression to provide profound insight into the transcriptional process by using RNA sequencing (RNA-Seq) [13]. In addition, the emergence of ribosome profiling (Ribo-Seq) offers an understanding of actual mRNA translation at a global scale. Ribo-Seq technology rescues ribosome-bound mRNA fragments from nuclease activity and measures the ribosome-protected mRNA fragments (RPFs), then calculates the abundance of the fragment to determine the rate of protein synthesis. Investigating translation leads to an enhanced perception of this energetically costly process in the cell, which is heavily regulated to economically allocate cellular resources. In turn, integration of the two omics data helps to untangle the complex relationship between transcription and translation, which enhances understanding of the genome to phenotype relationship [14, 15].

The aim of the present study was to unravel the systemic regulation of *E. limosum* ATCC 8486, a promising acetogenic bacteria, at the transcriptional and translational levels. To identify the differential gene expression and differential translation levels under heterotrophic and autotrophic conditions, we utilized RNA-Seq and Ribo-Seq, respectively, and then integrated the data to calculate translation efficiency (TE). Through integrated systemic analysis, we observed whether the translational regulation changes under the two different conditions, and assessed the relationship of such changes to the WLP and energy conservation system in the acetogenic bacterium.

## Results

### Determination of differential gene expression levels using RNA-Seq

The autotrophic condition triggers the expression of a wide array of genes related to acetogenesis at the levels of transcription and translation, which are subject to extensive regulation to ensure cell survival under autotrophy. To comprehend changes in gene expression levels of *E. limosum* ATCC 8486 under heterotrophic and autotrophic growth conditions, we first performed RNA-Seq. Total RNA samples were obtained from cells grown at mid-exponential phase in DSM 135 medium supplemented with glucose (5 g/L) or H_2_:CO_2_ (80:20 at 200 kPa) for heterotrophic or autotrophic condition, respectively. Maximal cell growth levels (optical density at 600 nm) of 2.00 ± 0.28 and 0.26 ± 0.01 were reached after 30 h and 118 h with 0.94 and 1.23 g/L of acetate formation as a main metabolic product under heterotrophic and autotrophic conditions, respectively. The obtained RNA samples were constructed into the sequencing libraries, which were subjected to deep sequencing. We obtained approximately 19.8 million sequence reads of high-quality, which were uniquely mapped to the genome sequence with an average read length of approximately 130 bp corresponding to 146.1-fold genomic coverage per each sample, according to the genome size of *E. limosum* ATCC 8486 of 4,422,837 bp [8].

**Fig. 1:**
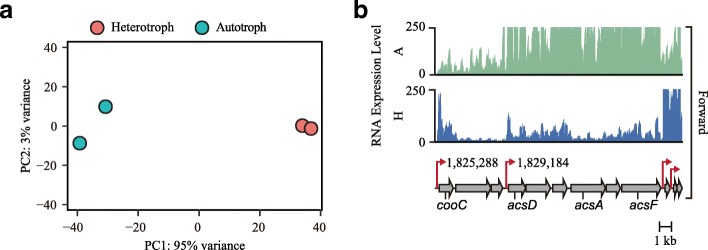
Transcriptome analysis under heterotrophic and autotrophic conditions. (**a**) Principal components analysis of the RNA-Seq data. Two distinct clusters based on heterotroph and autotroph growth conditions were weighted by variance (PC1: 95% variance) and change in expression over time (PC2: 3% variance). (**b**) Transcriptional expression of the gene cluster encoding the carbonyl branch of the Wood-Ljungdahl pathway (ELIM_c1647-ELIM_c1655). Red arrow indicates transcriptional start sites

RNA-Seq data were then normalized using the Bioconductor package DEseq2 [16]. Principal component analysis of the sequencing results demonstrated a significant difference in gene expression and a distinguishable expression profile between the two growth conditions (Fig. 1a). This indicated that the expression of a large array of genes in *E. limosum* ATCC 8486 is modulated for the temporal alteration of cellular functions between heterotrophic and autotrophic growth conditions. The normalized transcript abundance for each gene provides the differentially expressed genes (DEGs) between the two conditions with *P*-values adjusted for multiple testing using the Benjamini-Hochberg procedure [16]. The data produced using DEseq2 for all genes (total 4090 genes) are provided in Additional file 1: Table S1. The transcription of genes encoding the carbonyl branch of the WLP was highly upregulated under autotrophic growth conditions, indicating that the RNA-Seq data represents the activation of autotrophic metabolism under the conditions tested (Fig. 1b). We then obtained a list of DEGs between the two conditions that satisfied the criteria of at least a two-fold or greater change in gene expression and a *P*-value cutoff (DESeq *P*) < 0.05. A total of 1344 genes (31.4% of all genes) exhibited differential expression between the two conditions. These DEGs consisted of 664 upregulated and 680 downregulated genes, displaying a dynamic transcriptional range from − 273.39 to 217.55-fold change (Additional file 2**:** Table S2).

### Functional composition analysis of DEGs

To understand the cellular functions of DEGs, they were classified into the functional categories of the clusters of orthologous groups (COGs) along with their enrichment levels [17]. Among the DEGs, a total of 1269 genes were assigned to COGs, comprising 630 upregulated and 639 downregulated genes (Additional file 3**:** Table S3). COG analysis demonstrated that the genes belonging to “metabolism processes”, principally COG classes C, E, G, and H that refer to “energy production and conversion” (14.9%), “amino acid transport and metabolism” (8.9%), “carbohydrate transport and metabolism” (5.2%), and “coenzyme transport and metabolism” (5.9%), respectively, were mostly activated under the autotrophic growth conditions. These results are expected, as the cells were harvested under the autotrophic growth condition, which requires the involvement of a set of genes whose functions are associated with the WLP and Rnf complex for energy production and conversion. Accordingly, the relevant genes showed a significant upregulation under autotrophic growth conditions. In contrast, the genes related to “information storage and processing” and “cellular processes and signaling”, such as “transcription” (K, 13.6%) and “cell envelope and outer membrane biogenesis” (M, 6.6%), respectively, were largely downregulated under autotrophic growth conditions, consistent with the slower growth rate of the strain supplemented with CO_2_/H_2_ than that with glucose. The COG analysis revealed that the DEGs are involved in diverse biological processes and molecular functions, suggesting that the cellular responses to the autotrophic growth conditions are related to various metabolic processes.

**Fig. 2:**
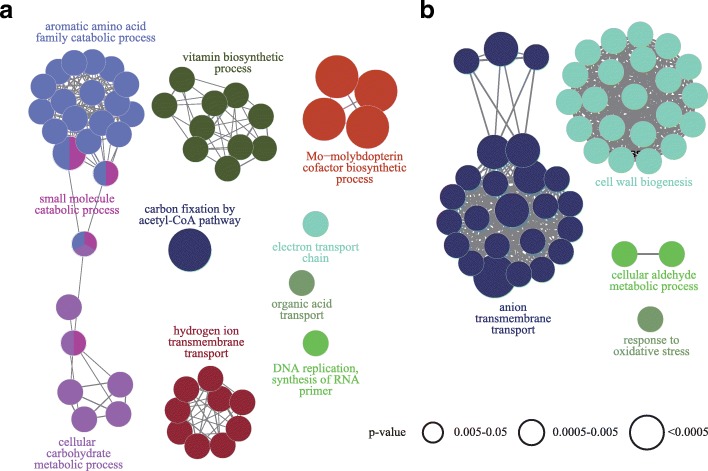
Significantly enriched KEGG pathways of the differentially expressed genes (DEGs). KEGG pathway enrichment of differently expressed genes; (**a**) upregulated DEGs. (**b**) downregulated DEGs. Each node represents a KEGG pathway and the node size is negatively correlated with the adjusted *P*-value of the pathway. An edge between two nodes denotes that the two pathways share common genes. The edge width is positively correlated with the number of common genes. Significant KEGG pathways of DEGs are classified into several functional groups based on κ value. Nodes of a group are labeled in the same color. The nodes of two colors are shared by two groups

Using Kyoto Encyclopedia for Genes and Genomes (KEGG) pathway enrichment analysis, we sought to identify which pathways were possibly involved in the obtained DEGs [18]. Collectively, 142 pathways were significantly enriched with the DEGs (Fig. 2a, b, and Additional file 4: Table S4), with 91 and 51 pathways functionally categorized into eight and four groups for the upregulated and downregulated DEGs based on the Kappa score (≥0.4), respectively. As expected, the pathways for the upregulated DEGs were primarily associated with autotrophic cell growth, such as “carbon fixation by acetyl-CoA pathway”, “electron transport chain”, “vitamin biosynthetic process”, “Mo-molybdopterin cofactor biosynthetic process”, and “hydrogen ion transmembrane transport”. Taken together, the increase in the mRNA transcript levels in these pathways emphasizes the importance of the Rnf complex, ATP synthase, cofactor biosynthesis-related genes, and WLP for cell growth under the autotrophic condition.

### Changes in expression levels of genes associated with WLP

Next, among the highly enriched metabolic pathways identified from KEGG pathway analysis, we elucidated the expression profiles of individual genes that are associated with central carbon metabolism and autotrophic growth, including genes encoding components of the methyl and the carbonyl branches of WLP, FDH, hydrogenase complexes, ATP synthase, and the Rnf complex.

**Fig. 3:**
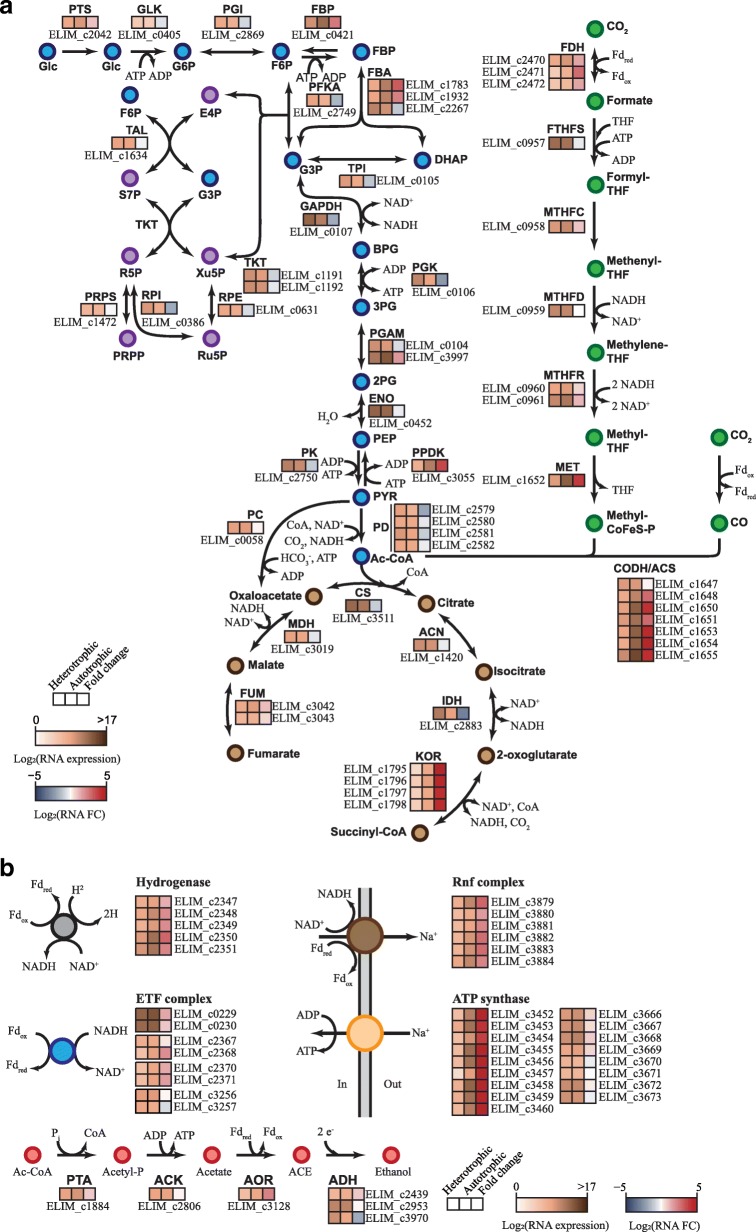
Different gene expression profiles in metabolic pathways of *E. limosum* ATCC 8486. (**a**) Embden-Meyerhof-Parnas glycolytic pathway (blue circle), incomplete tricarboxylic acid cycle (brown circle), Wood-Ljungdahl pathway (green circle), and pentose phosphate pathway (purple circle) are shown. (**b**) Ethanol producing pathway (red circle), hydrogenase complex (grey circle), Rnf complex (dark circle), ETF complex (blue circle), and ATP synthase complex (orange circle) are shown. Metabolites are shown as circles and reactions are shown as arrows. All gene numbers and enzyme reactions are provided next to the arrows. Transcriptional dynamics of *E. limosum* ATCC 8486 are described in the heatmap with three columns; the left and middle boxes indicate normalized RNA reads from heterotrophic and autotrophic conditions, respectively. The right box indicates RNA fold change, as autotrophic expression over heterotrophic expression. In addition, gene numbers responsible for the expression are located under the bottom box of the heatmap. A full list and expression data can be found in Additional file 1: Table S1

The reduction of CO_2_ to formate constitutes a ubiquitous metabolic reaction in many strict anaerobic microbes and the initial reaction of acetogenesis, which is catalyzed by FDH. The relevant genes are well conserved as core genes in all acetogenic bacteria [3, 9, 19]. In *E. limosum* ATCC 8486, an Fd-dependent FDH-encoding gene (ELIM_c2470) was annotated and located in the gene cluster with genes encoding molybdopterin-guanine dinucleotide biosynthesis protein (ELIM_c2471) and FDH-accessory protein (ELIM_c2472) [8]. All three genes were upregulated with a minimum fold change of > 2.84 (DESeq *P* < 2.15 × 10^− 6^) under the autotrophic growth condition (Fig. 3a and Additional file 5: Table S5). In turn, acetyl-CoA is produced from two CO_2_ molecules by a series of reactions catalyzed by the enzymes of the carbonyl and the methyl branches of the WLP [3, 20, 21]. In the methyl branch, the reduction of CO_2_ to formate by FDH is followed by a series of THF- and cobalamin-dependent reactions for the formation of a methyl group. In the carbonyl branch, the methyl group is condensed with CO (originating from another CO_2_) into acetyl-CoA by the enzyme complex carbon monoxide dehydrogenase/acetyl-CoA synthase (CODH/ACS) with coenzyme A (Fig. 3a). In contrast to other acetogenic bacteria such as *Clostridium ljungdahlii*, *C*. *aceticum*, and *C*. *autoethanogenum* [5, 7, 22], the gene clusters encoding the methyl and the carbonyl branches were located in different genomic regions in *E. limosum* ATCC 8486. Among five genes encoding the methyl branch (ELIM_c0957–ELIM_c0961), genes encoding formyl-THF cyclohydrolase, MTHFR, and methyltransferase were transcriptionally upregulated with a minimum fold change of > 1.53 (DESeq *P* < 3.28 × 10^− 3^) (Fig. 3a and Additional file 5: Table S5). Conversely, although *fhs* (ELIM_c0957) and *folD* (ELIM_c0959) exhibited high transcript levels under the two growth conditions (a minimum level of normalized sequence reads of > 584.23), their differential expression was not significant (DESeq *P* > 9.93 × 10^− 2^). Transcription levels of the genes encoding the carbonyl branch (ELIM_c1647–ELIM_c1655) were highly upregulated with a minimum fold change of > 7.30 (DESeq *P* < 1.84 × 10^− 11^) except for *cooC* (ELIM_c1647), which encodes a putative CODH nickel-insertion accessory protein under autotrophic growth conditions (Additional file 5: Table S5). CODH/ACS is encoded by *acsA* (ELIM_c1653) and *acsB* (ELIM_c1655), along with *acsC* (ELIM_c1650) and *acsD* (ELIM_c1651) for the corrinoid-iron sulfur protein. *acsE* (ELIM_c1652) transfers the methyl group from methyl-THF to the *acsCD* subunits of CODH/ACS. In addition, the genes *acsV* (ELIM_c1648) and *cooC* (ELIM_c1654), encoding a corrinoid activation/regeneration protein and a CODH nickel insertion accessory protein, respectively, were significantly upregulated with fold changes of > 7.31 (DESeq *P* < 1.84 × 10^− 11^) and > 33.59 (DESeq *P* < 8.65 × 10^− 37^). The gene cluster structure for the carbonyl branch is similar to that of *A. woodii* and *E. limosum* KIST 612 [6, 23]. Specifically, corrinoid iron-sulfur proteins play a key role in the transfer of the methyl group between methyltetrahydrofolate to a nickel center of ACS [24]. A total of six genes were predicted to encode corrinoid protein methyltransferases in *E. limosum* ATCC 8486. Among these, ELIM_c0445, ELIM_c0733, and ELIM_c1551 were highly upregulated with a minimum fold change of > 2.80 (DESeq *P* < 7.09 × 10^− 3^) (Additional file 5: Table S5). These results show good consistency with the transcription profiles of other acetogenic bacteria under autotrophic growth conditions, such as *C. ljungdahlii* and *C. autoethanogenum* [25, 26].

### Changes in expression levels of genes associated with energy conservation

WLP is tightly coupled with energy conservation systems. In acetogenic bacteria, the reduction of CO_2_ to formate catalyzed by the FDH is linked with the use of molecular hydrogen as an electron donor by hydrogenases. For example, *A. woodii*, an acetogenic bacterium closely related to *E. limosum* ATCC 8486, has a flavin-based electron-bifurcating hydrogenase, primarily for the reduction of one Fd and one NAD^+^ with two H_2_ [6]. Although the hydrogenase-encoding genes are not directly located adjacent to FDH-encoding genes as found in *A. woodii*, the genome of *E. limosum* ATCC 8486 encodes a gene cluster encoding putative hydrogenases (ELIM_c2347–ELIM_c2351) comparable to the bifurcating hydrogenase-encoding genes identified in *A. woodii* (*hydCEDBA*). Each subunit is similar to the corresponding one from *A. woodii* (Awo_c2347–Awo_c2351) with identities of 83.7% (HydC), 68.1% (HydE), 82.8% (HydD), 84.6% (HydB), and 79.4% (HydA). The putative hydrogenase-coding genes were transcriptionally activated with a minimum fold change of > 2.54 (DESeq *P* < 1.12 × 10^− 5^) (Fig. 3b and Additional file 6: Table S6).

Recently, it has been suggested that a bifurcating MTHFR with potential electron-transferring flavoproteins encoded by *etfAB* genes is plausible for reducing Fd as the electron donor for FDH function in *E. limosum* KIST612 [27]. Accordingly, two genes (ELIM_c0229 and ELIM_c0230) were found in *E. limosum* ATCC 8486 with upregulated transcription levels showing a fold change of > 2.78 (DESeq *P* < 2.08 × 10^− 5^) and > 1.88 (DESeq *P* < 1.49 × 10^− 2^), respectively (Fig. 3b and Additional file 6: Table S6). Notably, *metV* genes coding for MTHFR are conserved as dispensable genes in acetogenic bacteria, which demonstrates their enzymatic diversity. For example, a trimeric enzyme-complex was detected in *A. woodii*; however, a heterohexameric complex with an electron-bifurcating module was found in *M. thermoacetica* [7, 9]. Although the genes of redox enzymes were highly conserved, the configuration of enzymatic reactions may be different in acetogenic bacteria. Collectively, the transcriptional upregulation of genes encoding the putative flavin-based electron-bifurcating hydrogenase and the MTHFR coupled with electron-transferring flavoproteins indicates their potential role for the Fd-dependent reduction of CO_2_ in *E. limosum* ATCC 8486.

For ATP supply in acetogenic bacteria, an ion gradient needs to be established by membrane-bound protein complexes and then utilized by ATP synthase. In principle, ion-translocating protein complexes, either an Rnf or an Ech complex, accept a reduced Fd as an electron donor, and oxidation of the reduced Fd generates a membrane potential. In *E. limosum* ATCC 8486, an Rnf complex is encoded by a gene cluster (ELIM_c3879–ELIM_c3884) similar to the electron transfer system found in *A. woodii* and *E. limosum* KIST612 [6, 27]. However, genes encoding an Ech-type ion-translocating protein complex system were not detected in the *E. limosum* ATCC 8486 genome [8]. The transcription levels of the genes encoding the Rnf complex gene cluster were significantly increased under the autotrophic condition with a minimum fold change of > 3.30 (DESeq *P* < 6.13 × 10^− 7^) (Fig. 3b and Additional file 6: Table S6). Consistent with our observation, autotrophic-specific Rnf expression was detected in *C. autoethanogenum* [25]. Rnf function is also coupled with the aldehyde:Fd oxidoreductase (AOR) encoded by ELIM_c3128, which reduces acetate to aldehyde (Fig. 3b and Additional file 7: Table S7) [5, 28]. The *aor* gene was highly upregulated under autotrophic growth conditions with a fold change of > 5.11 (DESeq *P* < 1.18 × 10^− 9^).

Subsequently, a membrane-bound ATP synthase complex utilizes the ion-gradient generated by the Rnf complex. Most acetogenic bacteria contain the F-type ATP synthase complex; however, *E. limosum* contains only A/V-type ATP synthase complexes. Two ATP synthase clusters were annotated in the genome [8]. The first cluster (ELIM_c3452–ELIM_c3460) showed significant upregulation with a minimum fold change of > 15.04 (DESeq *P* < 7.62 × 10^− 35^) although the second ATP cluster (ELIM_c3666–ELIM_c3673) remained nearly unchanged (Fig. 3b, Additional file 1: Table S1, and Additional file 6: Table S6). These gene expression profiles indicate that the strain utilizes the first cluster to generate ATP under the autotrophic condition. Taken together, our findings indicate that the Rnf system and one of the two ATP synthase complexes provide a central pathway to generate ATP coupled with the reduction of Fd under autotrophic growth conditions.

### Changes in expression levels of genes associated with central carbon metabolism

Finally, genes composing central carbon metabolism proteins (i.e., glycolysis and the TCA cycle) were investigated (Fig. 3a and Additional file 7**:** Table S7). The major regulatory sites of glycolysis and gluconeogenesis comprise the phosphofructokinase (PFK) and fructose-1,6-bisphosphatase (FBP) catalyzed reactions. Under autotrophic growth conditions, ELIM_c2749 encoding PFK was downregulated with a fold change of > 2.53 (DESeq *P* < 1.31 × 10^− 4^) and ELIM_c0421 encoding FBP was highly upregulated with a fold change of > 5.74 (DESeq *P* < 4.90 × 10^− 14^). This indicates that glycolysis became repressed whereas gluconeogenesis was enforced under autotrophic growth conditions. Moreover, transcription levels of all genes responsible for glycolysis were downregulated or unchanged, whereas the genes encoding gluconeogenesis-related enzymes including FBP, fructose-bisphosphate aldolase (ELIM_c1783 and ELIM_1932), 2,3-bisphosphoglycerate-dependent phosphoglycerate mutase (ELIM_c3997), and pyruvate phosphate dikinase (ELIM_c3055) were upregulated with a minimum fold change of > 3.55 (DESeq *P* < 3.10 × 10^− 10^). Under autotrophic growth conditions, gluconeogenesis including the conversion of acetyl-CoA into pyruvate, and the TCA cycle plays key roles in providing precursors for the biosynthesis of nucleic acids, amino acids, and other metabolites in most acetogens [25, 29]. Although two putative gene clusters coding for pyruvate:ferredoxin oxidoreductase (PFOR, ELIM_c0697–ELIM_c0700, ELIM_c0874–ELIM_c0877) were found that catalyze the direct conversion of acetyl-CoA into pyruvate, notably, no associated gene expression was observed under either autotrophic or heterotrophic growth conditions (i.e., RPKM values between 0.00 and 2.45). Instead, we found a gene cluster annotated as 2-oxoacid:Fd oxidoreductase (KOR, ELIM_c1795–ELIM_c1798), which potentially catalyzes the reaction. These gene expression levels were highly upregulated under autotrophic growth conditions (a minimum fold change of > 19.77, DESeq *P* < 1.05 × 10^− 24^). In comparison, the TCA cycle was incomplete and forms a branched cycle to succinyl-CoA and fumarate (Fig. 3a). All relevant genes for the branched TCA cycle were downregulated or unchanged except the genes encoding for KOR.

We also identified genes for ethanol production from acetyl-CoA, specifically *pta* (ELIM_c1884), *ack* (ELIM_c2806), *aor* (ELIM_c3128), and *adh* (ELIM_c2439, ELIM_c2953, and ELIM_c3970) (Fig. 3a). Acetyl-CoA conversion to acetate regains one molecule of ATP by a substrate level phosphorylation catalyzed by PTA and ACK, which is expensed for the formation of formyl-THF from formate in the methyl branch [3, 20]. Notably, gene expressions of *ack* (ELIM_c2806) and *adh* (ELIM_c2953) were not upregulated or repressed by autotrophic growth although both genes showed high transcript levels under the two growth conditions (a minimum level of normalized sequence reads of > 816.45). However, *pta* and *aor* were upregulated for autotrophic growth (Additional file 7: Table S7). Taken together, the gene expression profiles suggest that *E. limosum* ATCC 8486 utilizes CO_2_ to form biomass and produce a wide array of metabolites using H_2_ as an electron donor via activated metabolic pathways including WLP, energy conservation systems, and gluconeogenesis, and represses glycolysis and the incomplete TCA cycle under autotrophic growth conditions.

### Determination of differential translational changes using ribosome profiling

Next, we questioned whether *E. limosum* ATCC 8486 synthesizes cellular proteins proportional to the changes in transcript levels between the two conditions. Conceptually, mRNA abundance is assumed to represent the concentration and activities of the corresponding proteins; however, their correlation is not strong (squared Pearson correlation coefficient of approximately 0.40) [30]. Numerous biological factors may cause this discrepancy, such as mRNA stability or post-transcriptional and translational regulation [31].

**Fig. 4:**
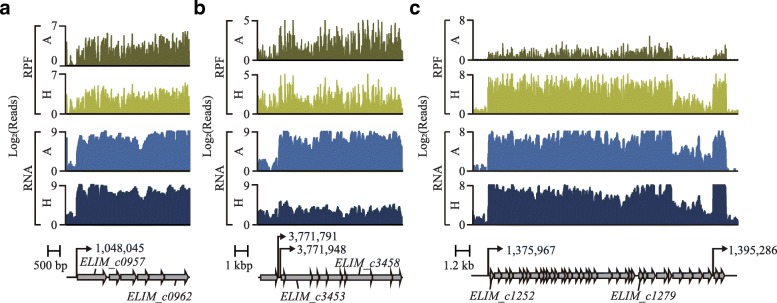
Different translation profiles of *E. limosum* ATCC 8486. Examples of transcription start sites, mRNA expression (RNA), and ribosome-protected fragment profiles (RPFs) at coding regions; (**a**) the methyl branch of the Wood-Ljungdahl pathway, (**b**) ATP synthesis cluster, and (**c**) ribosome biogenesis cluster. The transcription start sites are marked with black arrows

To address this question, we exploited the ribosome profiling approach, which provides the efficiency of translation initiation and elongation by sequencing RPFs to obtain insights into the regulation of protein abundance in cells [14]. High-throughput RPF sequencing resulted in more than 255 million uniquely mapped reads with an average read length of 32 bp. After normalization, the levels of unique mapped reads for each gene showed a high degree of correlation between biological replicates (Pearson correlation coefficient, *R* > 0.98). The evenly distributed RPF data, which represent the translational levels of each gene (Additional file 8: Table S8), were then compared with the transcriptome data, resulting in a positive correlation between transcription and translation levels for heterotrophic (R^2^ = 0.55, *P* < 5.05 × 10^− 219^) and autotrophic (R^2^ = 0.67, *P* < 4.26 × 10^− 269^) conditions (Additional file 9**:** Figure S1a and S1b). For example, the gene cluster (ELIM_c0957–ELIM_c0961) encoding the methyl branch of the WLP pathway showed similar changes in expression patterns between transcription and translation levels (Fig. 4a). Similarly, the changes in mRNA transcripts and RPF expression patterns were correlated in general, although genes associated with specific cellular functions exhibited the existence of regulation at the level of translation (i.e., poor correlation between the two levels). In particular, the gene cluster (ELIM_c3452–ELIM_c3460) encoding the ATP synthase showed apparently no changes in translation levels compared to the high degree of increase in mRNA transcript levels under autotrophic conditions (Fig. 4b). Furthermore, RPF expression of the ribosome biogenesis cluster (ELIM_c1252–ELIM_c1289) under the autotrophic condition showed fold changes less than − 2 with DESeq *P* < 4.92 × 10^− 17^, whereas transcription levels showed negligible changes (DEseq *P* > 0.05) or were upregulated (DEseq *P* > 1.25 × 10^− 4^) (Fig. 4c). This result is consistent with the regulation of ribosome biogenesis under energy-depleted conditions, which may affect TE owing to a limited number of ribosomal proteins being available [32].

### Changes in translation efficiency associated with autotrophic growth

**Fig. 5:**
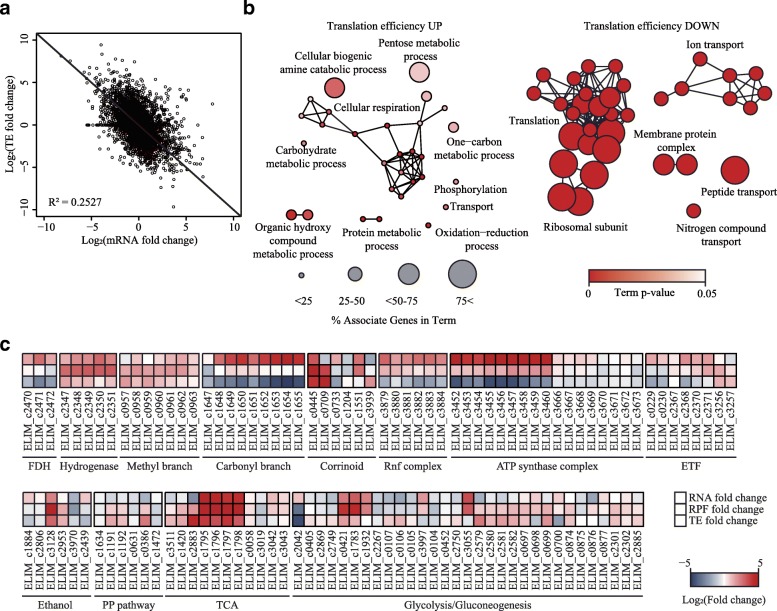
Translation efficiency (TE) of genes in *E. limosum* ATCC 8486. (**a**) A scatter plot of mRNA expression fold change (mRNA) and TE fold change under the heterotrophic condition and the autotrophic condition. (**b**) Annotation-term network of increased and decreased TE for “biological process”, “molecular function”, and “cellular component”. The node sizes represent associated gene percentages. The color indicates term enrichment significance. (**c**) A heatmap of RNA fold change (RNA FC), RPF fold change (RPF FC), and translation efficiency fold change (TE FC), from top to bottom. Full RPF and TE data can be found in Additional file 8: Table S8

We next calculated the TE of each gene by dividing RPF levels by the corresponding mRNA transcript levels (Additional file 8: Table S8). A negative correlation between mRNA fold change and TE, known as translational buffering, was observed between the two growth conditions (Fig. 5a) [33]. To understand which cellular functions are heavily regulated at the translational levels, functional networks of TE upregulated and downregulated genes were obtained as 33 GO terms enriched with 10 groups and 36 GO terms with 5 groups, respectively (Fig. 5b and Additional file 10: Table S9) [34]. “Translation”, “ion transport”, and “membrane protein complexes” were assigned to the TE downregulated genes. In comparison, cellular respiratory genes were assigned to the highly upregulated group along with the genes in the “biogenic amine catabolic process” and “oxidation-reduction process”.

Among the cellular respiratory systems, the RPF levels of transcriptionally upregulated genes encoding KOR (ELIM_c1795–ELIM_c1798), which was predicted to catalyze the direct conversion of acetyl-CoA into pyruvate with the reduction of Fd, were particularly upregulated with the lowest fold change of 227.61 (DESeq *P* < 1.29 × 10^− 200^) (Fig. 5c and Additional file 8: Table S8). This result strongly suggests that the required reduced electron carriers are obtained through activation of oxidoreductase genes under the autotrophic condition. Along with the reduction of electron carriers by the oxidoreductase genes, the hydrogenase gene clusters (ELIM_c2347–ELIM_c2351) are predicted to be responsible for the reduction for FDH via cofactors, in order to convert CO_2_ into formate. Similar to the expression pattern of KOR, the transcriptionally upregulated hydrogenase gene clusters exhibited upregulation of RPF levels by at least 7.74-fold (DESeq *P* < 1.09 × 10^− 35^). These results suggest that *E. limosum* ATCC 8486 allocates cellular energy to the enzyme complexes by activating transcriptional and translational expression, which indicates that hydrogenase plays an important role under the autotrophic condition.

Notably, in the case of the energy conservation system, RPF levels were negatively correlated with the amount of mRNA transcripts. The ATP synthase gene cluster (ELIM_c3452–ELIM_c3460), which is transcriptionally upregulated, showed significantly low TE levels, with the lowest fold change of 2.04 (DESeq *P* < 1.52 × 10^− 7^). In addition, similar low TE levels were also observed for the Rnf complex, which is responsible for oxidation of the reduced ferredoxin (Fig. 5c and Additional file 8: Table S8). The transcriptional abundance of the gene clusters encoding the carbonyl branch of WLP and FDH was upregulated, although strikingly, none of the genes were significantly changed in RPF expression and showed low TE under autotrophic conditions (Fig. 5c). All prior transcriptome studies, including the present study, have reported that the genes encoding the carbonyl branch were significantly upregulated, whereas this ribosome profiling result provides critical evidence that the cluster is translationally regulated [25, 26, 35].

**Fig. 6:**
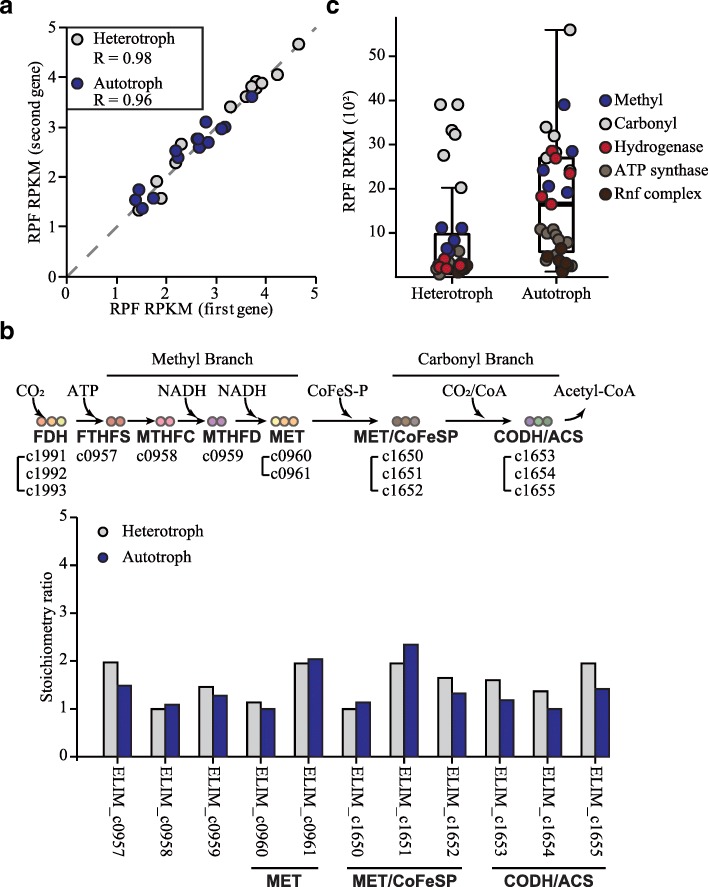
Stoichiometry ratio and synthesis rate of protein complex subunits in *E. limosum*. (**a**) Correlation of RPF expressions between first and second genes in the corresponding operon, which responsible for translating subunits of protein complexes (Additional file 11: Table S10). All of the subunits are known to be composed of one to one stoichiometric ratio. Grey circle indicates *E. limosum* cultured under the heterotrophic condition and blue circle indicates the cell cultured under the autotrophic condition. (**b**) Stoichiometry ratio of the proteins in methyl and the carbonyl branch of WLP. The grey circle indicates the ratio under the heterotrophic condition and the blue circle indicates the ratio under the autotrophic condition. (**c**) RPF expression of genes associated with the methyl and the carbonyl branch of WLP, hydrogenase complex (ELIM_c2347-ELIM_c2351), ATP synthase complex (ELIM_c3452-ELIM_c3460), and Rnf (ELIM_c3879-ELIM_c3884) that colored by blue, grey, red, light brown, and dark brown, respectively

To understand the translational regulation of WLP, stoichiometric synthesis of protein complex subunits were examined using the Ribo-Seq results. First, 59 subunits, which reported to be composed of stoichiometric ratio of one to one, demonstrate that the expressions of neighbor genes were well conserved with Pearson correlation coefficient of 0.98 and 0.96 for the heterotrophic and the autotrophic condition, respectively (Fig. 6a and Additional file 11: Table S10) [36]. Then, the protein subunits composing the methyl and the carbonyl branch were investigated. The ratio of the protein complex associated with the methyl branch was maintained to be proportional to their stoichiometry ratio under the both conditions, which consistent with the previous studies (Fig. 6b) [37–39]. In contrast, the ratio between the complexes associated with the carbonyl branch also remained [40, 41]. The synthesis rates of the methyl branch and hydrogenase coding genes, under the heterotrophic condition, were lower than those under the autotrophic condition (Fig. 6c). However, the synthesis rates of the methyl branch enzymes and hydrogenase under the autotrophic condition were similar to the rates of the carbonyl branch. Such results suggest that the transcriptionally activated genes encoding the carbonyl branch were translationally regulated to maintain equal-molar ratio between the methyl and the carbonyl branch protein complex under the energy depleting condition. Overall, the integrated analysis of the two datasets provided direct evidence that under the autotrophic condition, the acetogenic bacteria, at the translation level, controls energy conservation and WLP.

A cause of the post-transcriptional regulation on the genes encoding the WLP and the energy conservation system is unclear. One possible explanation is protein complexes, which translation efficiency decreased, may be associated with their cellular location. Such physical limitation may limit translation of membrane-associated proteins under cell growth. Of post-transcriptionally regulated complexes, Rnf and ATP synthase complexes are well known trans-membrane proteins, but whether CODH in *E. limosum* interacts with membrane is unknown. In acetogens, such as *Carboxydothermus hydrogenoformans* and *Moorella thermoacetica*, CODH associates with inner cytoplasmic membrane [4, 42, 43]. Certainly, further study is needed to validate the hypothesis to understand the regulation on translation of the systems that reported to play essential roles in acetogenesis.

### Analysis of regulatory features within the 5′UTR

We next attempted to evaluate the TE results of the carbonyl branch of WLP, which was inefficiently translated under the autotrophic condition. Toward this end, a β-galactosidase reporter system was constructed in a plasmid bearing 300 bp upstream sequence of the responsible transcription start site (TSS), the 5′UTR, and the *lacZ* gene. 5′UTR regions of the carbonyl branch genes and enoyl-CoA hydratase gene (ELIM_c1491) were selected for the evaluation of TE, using the recently determined genome-wide information of TSSs [8]. The constructed plasmids were transformed into *E. limosum* ATCC 8486, then cultured until the same sampling time point as in RNA-Seq and Ribo-Seq under the same conditions. The transcript levels of the *lacZ* gene controlled by the inserted promoter sequences were measured using reverse transcription-PCR. The fold changes of *lacZ* mRNA expression under promoters of ELIM_c1647, ELIM_c1650, and ELIM_c1491 between the autotrophic and the heterotrophic conditions were 1.01, 20.91, 2.75, respectively, which were highly correlated with RNA-Seq results (R^2^ = 0.99). β-galactosidase expression was also assayed to measure the translation levels, which resulted in the fold change of 0.62, 0.99, and 3.90, respectively. The relative TE between the conditions were then calculated to be 0.61, 0.05, and 1.42, respectively, validating the results obtained from the ribosome profiling with repression of WLP-associated UTRs and activation of enoyl-CoA hydratase associated UTRs (Additional file 12: Figure S2a). Thus, the carbonyl branch of WLP is tightly regulated at the translational level.

The 5′UTR, which is completely untranslated, instead forming a complex secondary structure, plays an important role for the regulation of translation. To identify the relationship between the 5′UTR and TE, 1223 secondary structures were derived from the identified 5′UTRs based upon the previously determined TSSs [8]. Among these, 285 TE upregulated genes and 347 TE downregulated genes were selected for comparison. The difference between heterotrophic and autotrophic free energy of the 5′UTR was highly significant (*P* = 3.27 × 10^− 4^) with median values of − 2.1 kcal/mol and − 3.4 kcal/mol for increased and decreased TE, respectively (Additional file 12: Figure S2b). For decreased TE transcripts, an altered ribosome footprint distribution at the 5′UTR was observed with significant difference (*P* = 1.44 × 10^− 2^) under heterotrophic and autotrophic conditions (Additional file 12: Figure S2c), whereas insignificant change was observed (*P* = 1.43 × 10^− 1^) for total TE transcripts (Additional file 12: Figure S2d). A similar pattern was observed in many decreased TE transcripts including genes encoding an ATP synthase subunit (ELIM_c3460) and ribosomal protein biogenesis (ELIM_c1252) (Additional file 12: Figure S2e and S2f). 5′UTR structure for both transcripts showed low free energies, − 83.4 and − 5.0 kcal/mol, respectively with significant differences (*P* = 1.99 × 10^− 3^ and *P* = 3.23 × 10^− 2^) between the two conditions. Notably, with a long 5′UTR length (129 nt) and low free energy (− 20.6 kcal/mol), the genes encoding the carbonyl branch tended to be downregulated under the autotrophic condition with a minimum fold change of 3.16. In contrast, the free energy of the second part of the methyl branch gene cluster was higher (− 2.3 kcal/mol) than those of other genes described above. Taken together, the integration of RNA-Seq and Ribo-Seq data supports that TE of mRNA is partly regulated by 5′UTR structure with downregulated translation of ribosomal proteins under the energy limited conditions and relatively less translation energy available at the cellular level.

## Discussion

In the present study, we determined the transcriptome and translatome using RNA-Seq and Ribo-Seq, respectively, to systematically understand the regulation of gene expression associated with syngas fermentation metabolism in *E. limosum* ATCC 8486. With 4090 protein coding genes in total, the transcriptome analysis of *E. limosum* ATCC 8486 showed significant upregulation of the WLP, hydrogenase complex, Rnf complex, ATP synthase complex, and gluconeogenesis under autotrophic conditions [25, 26]. Along with upregulation of FDH, the hydrogenase complex, which was predicted to be responsible for the initial step of WLP, showed significant upregulation of transcription. However, the significant increase in mRNA abundance of genes encoding the Rnf complex with a minimum fold change of 3.30 (DESeq *P* < 6.13 × 10^− 7^) was not correlated with the transcriptome analysis of *C. ljungdahlii*, in which insignificant changes were determined [26, 35]. Such results emphasize that phylogenetically diverse acetogens maintain different molecular mechanisms for syngas fermentation. In addition, based on the ribosome profiling data, we determined a reduction of translation rate in the carbonyl branch of the WLP and energy conservation system under the autotrophic condition. The stoichiometric synthesis ratio between the two branches indicated that the protein subunits of the translationally regulated carbonyl branch remained to be equal-molar to the subunits of the methyl branch. The result suggests that *E. limosum*, under autotrophic growth, tightly regulates translation of WLP associated genes, specifically the carbonyl branch coding genes, to efficiently form the protein complex under energy depleted condition. From the decreased TE genes, stable 5′UTR secondary structures were identified, which likely requires higher levels of GTP to break the structure for elongation under the energy depleted autotrophic condition [44, 45].

Under the autotrophic condition, the TEs of ribosome biogenesis and of all translation initiation factors (ELIM_c1277, ELIM_c3731, and ELIM_c3488) were decreased. With translationally regulated ribosome biosynthesis, freely available ribosome complexes for translation should decrease in a cell, which would heavily influence the translation of mRNA and thereby alter proteome allocation for the cellular economy via translational regulation [46, 47]. Despite these complex relationships, the massive upregulation at the transcription level and decreased TE of WLP-associated genes raises questions regarding their regulatory networks under the autotrophic condition. This observation suggests that the acetogenic bacteria allocate cellular resources properly, focusing more on the translation of energy conservation system rather than on carbon metabolism genes. Such examples include genes encoding cellular respiratory and electron bifurcation, such as KGOR, IOR, AOR, and the hydrogenase complex. Significant upregulation of the RPF and TE of these genes verifies the critical roles of the oxidoreductases as electron carriers in energy conservation under the autotrophic condition [48]. Together with these results, the transcriptional and translational information will likely provide an important resource for understanding syngas fermentation. In addition, we suggest that energy metabolism is essential, even to a greater degree than carbon fixation, under the autotrophic condition and may constitute a potential target for strain engineering for obtaining better productivity.

## Conclusion

In this study, by integrating transcriptomic and translatomic data, we identified transcriptionally abundant genes associated with the carbonyl branch of WLP, Rnf complex, and ATP synthase complex, showed decreased TE, whereas genes encoding the methyl branch of WLP, cellular respiration, and electron bifurcation exhibited highly enhanced TE. The finding indicates *E. limosum*, using regulatory features in 5′UTR, reallocates protein synthesis and energy economically, focusing more on the translation of genes for the generation of reduced electron carriers via electron bifurcation, rather than on those for carbon metabolism under autotrophic growth.

## Methods

### Bacterial strains and growth conditions

*E. limosum* ATCC 8486 was obtained from the Leibniz Institute DSMZ-German Collection of Microorganisms and Cell Cultures (DSMZ, Braunschweig, Germany) and cultivated anaerobically at 37 °C in 100 mL DSM 135 medium, which composed of 18.7 mM NH_4_Cl, 2.4 mM KH_2_PO_4_, 2.6 mM K_2_HPO_4_, 0.4 mM MgSO_4_ × 7 H_2_O, 0.2% (*w*/*v*) yeast extract, 119.0 mM NaHCO_3_, 2.8 mM L-cysteine-HCl × H_2_O, 4 μM resazurin, 20 mL trace element solution (DSMZ, media 141), and 10 mL vitamin solution (DSMZ, media 141) per liter medium, supplemented with 5 g/L glucose or H_2_/CO_2_ (80:20) at a pressure of 200 kPa with 50 mL of headspace for the heterotrophic and the autotrophic growth conditions, respectively (see DSMZ, media 135 for more detail). The cultured cells were harvested by anaerobic centrifugation at 4000 *g*, washed with basal DSM 135 medium, and inoculated in 100 mL of new medium with 50 mL of headspace, adjusting initial optical density 600 nm to be around 0.05. For RNA-Seq and Ribo-Seq, samples were harvest in mid-exponential phase at optical density 600 nm of around 0.80 and 0.20 for heterotrophic and autotrophic conditions, respectively, which growth measurement can be found in the previous paper [8].

### RNA-Seq

The biological duplicate cells were resuspended in 500 μL lysis buffer composed of 20 mM Tris-HCl (pH 7.4), 140 mM NaCl, 5 mM MgCl_2_, and 1% Triton X-100. Resuspended cells were frozen in liquid nitrogen and then ground with pestle and mortar. The powdered cells were thawed and the cell debris was removed by centrifugation at 4000 *g* for 15 min at 4 °C. Total RNA was then isolated using TRIzol (Thermo Scientific). To remove residual genomic DNA in the RNA sample, 4 U of rDNase I (Ambion) was applied to the isolated RNA for 1 h at 37 °C, followed by incubation at 75 °C for 10 min. Ribosomal RNAs (rRNAs) were removed by using the Ribo-Zero™ rRNA Removal Kit for Meta-bacteria (Epicentre), and the rRNA-depleted RNA quality was checked using the Agilent 2200 TapeStation system (Agilent Technologies). Using the TruSeq Stranded mRNA Library Prep Kit (Illumina), the rRNA-depleted RNAs were converted into RNA-Seq libraries, which were then sequenced by the 150 bp read recipe with an Illumina Miseq™ system.

### Ribosome profiling (Ribo-Seq)

For Ribo-Seq, 100 μM chloramphenicol (CM) was added to the biological duplicate cultures which were then further incubated at 37 °C for 10 min. The CM treated cells were subsequently washed using 500 μL polysome buffer composed of 20 mM Tris-HCl (pH 7.4), 140 mM NaCl, 5 mM MgCl_2_, and 100 μM CM, and resuspended in lysis buffer composed of 20 mM Tris-HCl (pH 7.4), 140 mM NaCl, 5 mM MgCl_2_, 100 μM CM, and 1% Triton X-100. The resuspended cells were frozen in liquid nitrogen and ground with pestle and mortar. The powdered cells were recovered by centrifugation at 4000 *g* for 15 min at 4 °C, then the supernatant was additionally centrifuged at 16,000 *g* for 10 min at 4 °C. To degrade RNA that was unprotected by ribosomes, 400 U MNase (NEB), 2 μL bovine serum albumin (1 mg/mL), and 20 μL of 10× MNase buffer were added and incubated at 37 °C for 2 h with gentle rotation. For inactivation of the reaction, 10 μL EGTA (0.5 M) was added to the sample. The monosome fraction was recovered using Microspin S-400 HR columns (GE). The recovered ribosome-bound RNA was isolated by using TRIzol and the remaining rRNAs were removed with the Ribo-Zero™ rRNA Removal Kit for Meta-bacteria. For the phosphorylation reaction, samples were denatured at 80 °C for 90 s, equilibrated to 37 °C, and incubated at 37 °C for 1 h with 5 μL of 10× T4 PNK buffer (NEB), 20 U SUPERase-In RNase Inhibitor, and 10 U T4 PNK (NEB). After purification of the RNA samples using an RNeasy MinElute Column (Qiagen), the concentration of purified RNA was measured using the Qubit RNA HS assay kit (Invitrogen). For library construction, the small RNA library prep kit for Illumina (NEB) was used and the constructed library was sequenced using the 50 bp read recipe on an Illumina Hiseq2500.

### Data processing

The sequence reads were trimmed to remove the adapter sequences. Using CLC Genomics Workbench, the trimmed reads were aligned to the assembled *E. limosum* ATCC 8486 genome with following parameters: mismatch cost = 2, deletion cost = 3, insertion cost = 3, length fraction = 0.8, and similarity fraction = 0.8; and the uniquely mapped reads were retained [8]. For RNA-Seq, the gene expression levels were normalized using DESeq2 package in R with default parameters [16]. For Ribo-Seq, after trimming the adapter sequences, reads with shorter than 20 bp were removed. The trimmed reads were aligned to the genome and normalized as for RNA-Seq. For stoichiometry calculation,

### Quantitative real-time polymerase chain reaction (qPCR)

The same RNA samples used for RNA-Seq library preparation were converted to cDNA using the SuperScript III First-Strand Synthesis System (Invitrogen) in accordance with manufacturer instruction. The reaction was performed using SYBR FAST qPCR master mix (KAPA BIO) and monitored on a CFX96™ Real-Time PCR Detection System (Bio-Rad) under the following conditions: 98 °C for 10 s; 62 °C for 30 s; 72 °C for 10 s for 40 cycles. The results were normalized with guanylate kinase (ELIM_c1967) and formate-tetrahydrofolate ligase (ELIM_c0957) as controls. The sequences of primers can be provided upon request.

### Plasmid construction

*Escherichia coli* strain NEB Express was used for plasmid propagation and cloning. Plasmid for electroporation into *E. limosum* was isolated from *E. coli* strain ER2275 to allow in vivo methylation. *E. coli* was cultivated in LB medium at 37 °C. For DNA amplification, Pfu-X polymerase (Solgent) was used. *E. limosum* genomic DNA was used for amplification of target promoter sequences and the *lacZ* gene was obtained from *E. coli* MG1655 gDNA. All of the primers are indicated in Table S2, which used to amplify at following condition: 95 °C for 2 min; 95 °C for 20 s; 60 °C for 30 s; 72 °C for 30 s/Kb for 35 cycles; 72 °C for 5 min. Plasmids were constructed by inserting the amplicons into the pJIR750ai plasmid (Sigma-Aldrich) by using restriction enzyme sites AclI and SapI and ligated using T4 DNA ligase.

### Electrotransformation

Electrotransformation was conducted as previously described [49]. All of the experiments were carried out in an anaerobic chamber. *E. limosum* were cultured in 100 mL DSM 135 medium supplemented with 5 g/L glucose. At the mid-exponential phase, the cell pellet was obtained via centrifugation for 15 min at 10,000 *g*. The collected cell pellet was washed twice with 100 mL sucrose buffer (270 mM) and resuspended to a final concentration of 10^11^ cells/mL, then transferred to a 0.1-cm-gap Gene Pulser cuvette (Bio-Rad). Then, 1 μg plasmid was added to the prepared cells and pulsed at 0.63 kV. Immediately following, the cells were recovered using 1 mL reinforced clostridial medium (RCM) and incubated at 37 °C until clear growth was observed (6 to 8 h). The recovered cells were plated on an RCM plate (1.5% agar) containing an appropriate antibiotic. A single colony was selected and cultured in DSM 135 medium supplemented with 5 g/L glucose for proliferation, then confirmed via plasmid DNA isolation using a DNA-spin™ plasmid DNA purification kit (iNtRON).

### β-Galactosidase activity

At the sampling time point, the cell culture was centrifuged at 10,000 *g* at 4 °C for 15 min and washed twice with phosphate buffered saline (1.7 mM KH_2_PO_4_, 5 mM NaHPO_4_, and 150 mM NaCl pH 7.4). The pellet was resuspended in 500 mL Tris Buffer (0.25 M), pH 8.0 and the solution ground using liquid nitrogen in a mortar. Aliquots of 1, 5, and 10 μL lysate were transferred to new microcentrifuge tubes and the final volume adjusted to 30 μL, to which 70 μL ortho-nitrophenyl-β-galactosidase and 200 mL cleavage buffer (60 mM Na_2_HPO_4_-7H_2_O, 40 mM NaH_2_PO_4_-H_2_O, 10 mM KCl, and 1 mM MgSO_4_-7H_2_O at pH 7.0) were added, then mixed by flicking the tube. The reacted solution was incubated at 37 °C for 30 min, and then 500 μL Na_2_CO_3_ (1 M) was added to terminate the reaction. The solution intensity was measured at an absorbance of 420 nm.

## Additional files

Additional file 1: **Table S1.** Transcription expression of genes in *Eubacterium limosum* under heterotrophic and autotrophic growth conditions (XLSX 552 kb)

Additional file 2: **Table S2.** Significantly regulated genes of *Eubacterium limosum* in response to the autotrophic growth condition (XLSX 105 kb)

Additional file 3: **Table S3.** COG functional assignments of differently expressed genes (DEGs) in *Eubacterium limosum* between heterotrophic and autotrophic growth conditions (DOCX 17 kb)

Additional file 4: **Table S4.** KEGG pathways sorted by transcriptionally UP and DOWN regulated genes in *Eubacterium limosum* under heterotrophic and autotrophic growth conditions (XLSX 18 kb)

Additional file 5: **Table S5.** Transcription profile of genes associated with the Wood-Ljungdahl pathway (DOCX 17 kb)

Additional file 6: **Table S6.** Transcription profile of genes associated with energy conservation (DOCX 17 kb)

Additional file 7: **Table S7.** Transcription profile of genes associated with central carbon metabolism (DOCX 20 kb)

Additional file 8: **Table S8.** Ribosome profiling analysis of genes in Eubacterium limosum under heterotrophic and autotrophic growth conditions (XLSX 537 kb)

Additional file 9: **Figure S1.** Translatome analysis of *E. limosum* ATCC 8486. (**a**) Comparison between RNA-Seq and Ribo-Seq under heterotrophic condition. Scatter plot between ribosome-protected fragment profile (RPF) as the y-axis and mRNA expression (mRNA) as the x-axis. (**b**) Comparison between RNA-Seq and Ribo-Seq under autotrophic conditions. The results show positive correlation with R^2^ of 0.55 and 0.67, respectively. (TIF 1104 kb)

Additional file 10: **Table S9.** KEGG pathways sorted by translation efficiency UP and DOWN regulated genes in *Eubacterium limosum* (XLSX 18 kb)

Additional file 11: **Table S10.** Protein subunits of known protein complexes used in stoichiometry analysis (XLSX 14 kb)

Additional file 12: **Figure S2.** Analysis of translational regulation with determined 5′UTRs. (**a**) The relative mRNA expression, protein expression, and TE values of ELIM_c1647 (c1647_*lacZ*), ELIM_c1650 (c1650_*lacZ*), and ELIM_c1491 (c1491_*lacZ*) between autotrophic and heterotrophic conditions. TE values were calculated by dividing the relative protein expression obtained from β-galactosidase assay by relative mRNA expression. (**b**) Box plot showing ΔG (kcal/mol) values of UTRs with median value of − 2.1 and − 3.5 kcal/mol for TE upregulated and downregulated genes, respectively. (**c**-**f**) Ribosome coverage across the 5’UTR, coding region, and 3’UTR for decreased TE transcript (**c**), a total of 1223 genes (**d**), the ATP synthase cluster transcript (**e**), and the ribosomal protein biogenesis transcript (**f**) under the heterotrophic and the autotrophic conditions. The dotted lines indicate translation start and stop sites. The black and red lines represent the ribosome coverage under the heterotrophic and autotrophic conditions, respectively. *P < 0.05; **P < 0.01; ***P < 0.001 (Wilcoxon rank-sum test). (TIF 1563 kb)

## Abbreviations

COGs: clusters of orthologous groups
DEGs: differentially expressed genes
KEGG: Kyoto Encyclopedia for Genes and Genomes
Ribo-Seq: ribosome profiling sequencing
RNA-Seq: RNA sequencing
RPFs: ribosome-protected mRNA fragments
TE: translation efficiency
