# Supplementary material for: Genome-scale analysis of syngas fermenting acetogenic bacteria reveals the translational regulation for its autotrophic growth
Source: BMC Genomics. 2018 Nov 23;19:837. doi: 10.1186/s12864-018-5238-0 (PMC6260860; doi:10.1186/s12864-018-5238-0)
Supplement: Supplementary file 3 — Table S3. COG functional assignments of differently expressed genes (DEGs) in Eubacterium limosum between heterotrophic and autotrophic growth conditions (DOCX 17 kb) [file 12864_2018_5238_MOESM3_ESM.docx]

**Table S3.** COG functional assignments of differently expressed genes (DEGs) in *Eubacterium limosum* between heterotrophic and autotrophic growth conditions

| **COG Class** | **Description** | **Enrichment** | | | |
| --- | --- | --- | --- | --- | --- |
|  |  | **Upregulated genes** | | **Downregulated genes** | |
|  |  | CDS | %^a^ | CDS | %^a^ |
| C | Energy production and conversion | 94 | 14.9 | 31 | 4.9 |
| D | Cell cycle control, cell division, chromosome partitioning | 4 | 0.6 | 4 | 0.6 |
| E | Amino acid transport and metabolism | 56 | 8.9 | 39 | 6.1 |
| F | Nucleotide transport and metabolism | 6 | 1.0 | 13 | 2.0 |
| G | Carbohydrate transport and metabolism | 33 | 5.2 | 31 | 4.9 |
| H | Coenzyme transport and metabolism | 37 | 5.9 | 20 | 3.1 |
| I | Lipid transport and metabolism | 11 | 1.7 | 10 | 1.6 |
| J | Translation, ribosomal structure and biogenesis | 26 | 4.1 | 20 | 3.1 |
| K | Transcription | 38 | 6.0 | 87 | 13.6 |
| L | Replication, recombination and repair | 26 | 4.1 | 23 | 3.6 |
| M | Cell wall/membrane/envelope biogenesis | 8 | 1.3 | 42 | 6.6 |
| N | Cell motility | 2 | 0.3 | 4 | 0.6 |
| O | Posttranslational modification, protein turnover, chaperones | 21 | 3.3 | 18 | 2.8 |
| P | Inorganic ion transport and metabolism | 32 | 5.1 | 34 | 5.3 |
| Q | Secondary metabolites biosynthesis, transport and catabolism | 11 | 1.7 | 3 | 0.5 |
| S | Function unknown | 184 | 29.2 | 207 | 32.4 |
| T | Signal transduction mechanisms | 25 | 4.0 | 28 | 4.4 |
| U | Intracellular trafficking, secretion, and vesicular transport | 5 | 0.8 | 4 | 0.6 |
| V | Defense mechanisms | 11 | 1.7 | 21 | 3.3 |

^a^ The enrichment level (%) was defined as the ratio between the number of genes in the COG class and the total upregulated (630) and downregulated (639) DEGs, respectively.
