# Supplementary material for: Genome-scale analysis of syngas fermenting acetogenic bacteria reveals the translational regulation for its autotrophic growth
Source: BMC Genomics. 2018 Nov 23;19:837. doi: 10.1186/s12864-018-5238-0 (PMC6260860; doi:10.1186/s12864-018-5238-0)
Supplement: Supplementary file 5 — Table S5. Transcription profile of genes associated with the Wood-Ljungdahl pathway (DOCX 17 kb) [file 12864_2018_5238_MOESM5_ESM.docx]

**Table S5.** Transcription profile of genes associated with the Wood-Ljungdahl pathway

| **Locus Tag** | **Gene** | **Description** | **FC (log2)** | ***P*-value** |
| --- | --- | --- | --- | --- |
| ELIM_c2470 | *fdhA* | NAD-dependent formate dehydrogenase | 1.51 | 2.15 × 10^-6^ |
| ELIM_c2471 | *mobA* | Molybdopterin-guanine dinucleotide biosynthesis protein A | 2.85 | 1.09 × 10^-11^ |
| ELIM_c2472 | *fdhD* | FdhD protein | 1.52 | 3.16 × 10^-4^ |
| ELIM_c0957 | *fhs* | Formyl-THF synthetase | -0.31 | 3.07 × 10^-1^ |
| ELIM_c0958 | *fchA* | Formyl-THF cyclohydrolase | 0.91 | 3.28 × 10^-3^ |
| ELIM_c0959 | *folD* | Methylene-THF dehydrogenase | 0.00 | 9.91 × 10^-1^ |
| ELIM_c0960 | *metV* | Methylene-THF reductase | 0.62 | 9.94 × 10^-2^ |
| ELIM_c0961 | *metF* | Methyltransferase | 1.20 | 2.11 × 10^-4^ |
| ELIM_c0962 | *lpdA* | Dihydrolipoamide dehydrogenase | 1.49 | 3.40 × 10^-6^ |
| ELIM_c0963 | *gcvH* | Glycine cleavage system H protein | 0.69 | 4.18 × 10^-2^ |
| ELIM_c1647 | *cooC* | CODH nickel-insertion accessory protein | 0.12 | 7.33 × 10^-1^ |
| ELIM_c1648 | *acsV* | Corrinoid activation/regeneration protein | 2.87 | 1.84 × 10^-11^ |
| ELIM_c1649 |  | Hypothetical protein | 3.78 | 4.05 × 10^-21^ |
| ELIM_c1650 | *acsD* | Corrinoid-iron sulfur protein | 4.24 | 9.42 × 10^-27^ |
| ELIM_c1651 | *acsC* | Corrinoid-iron sulfur protein | 2.89 | 5.21 × 10^-13^ |
| ELIM_c1652 | *acsE* | Methyltransferase | 4.00 | 3.73 × 10^-29^ |
| ELIM_c1653 | *acsA* | CO dehydrogenase/acetyl-CoA synthase subunit beta | 4.49 | 1.72 × 10^-31^ |
| ELIM_c1654 | *cooC* | CODH nickel-insertion accessory protein | 5.07 | 8.65 × 10^-37^ |
| ELIM_c1655 | *acsB* | Acetyl-CoA synthase | 4.32 | 8.51 × 10^-30^ |
| ELIM_c0445 | *mttB* | Corrinoid protein methyltransferase | 3.41 | 7.69 × 10^-7^ |
| ELIM_c0709 | *mttB* | Corrinoid protein methyltransferase | -1.18 | 5.31 × 10^-3^ |
| ELIM_c0733 | *mttB* | Corrinoid protein methyltransferase | 1.49 | 7.09 × 10^-3^ |
| ELIM_c1204 | *mttB* | Corrinoid protein methyltransferase | -1.10 | 1.84 × 10^-2^ |
| ELIM_c1551 | *mttB* | Corrinoid protein methyltransferase | 2.75 | 3.79 × 10^-5^ |
| ELIM_c3939 | *mttB* | Corrinoid protein methyltransferase | -1.96 | 2.11 × 10^-2^ |
