# Supplementary material for: Genome-scale analysis of syngas fermenting acetogenic bacteria reveals the translational regulation for its autotrophic growth
Source: BMC Genomics. 2018 Nov 23;19:837. doi: 10.1186/s12864-018-5238-0 (PMC6260860; doi:10.1186/s12864-018-5238-0)
Supplement: Supplementary file 6 — Table S6. Transcription profile of genes associated with energy conservation (DOCX 17 kb) [file 12864_2018_5238_MOESM6_ESM.docx]

**Table S6.** Transcription profile of genes associated with energy conservation

| **Locus Tag** | **Gene** | **Description** | **FC (log2)** | ***P*-value** |
| --- | --- | --- | --- | --- |
| ELIM_c3879 | *rnfB* | Electron transport complex protein RnfB | 3.03 | 6.83 × 10^-20^ |
| ELIM_c3880 | *rnfA* | Electron transport complex protein RnfA | 1.72 | 6.13 × 10^-7^ |
| ELIM_c3881 | *rnfE* | Electron transport complex protein RnfE | 2.23 | 3.43 × 10^-10^ |
| ELIM_c3882 | *rnfG* | Electron transport complex protein RnfG | 2.82 | 1.23 × 10^-20^ |
| ELIM_c3883 | *rnfD* | Electron transport complex protein RnfD | 2.86 | 3.56 × 10^-20^ |
| ELIM_c3884 | *rnfC* | Electron transport complex protein RnfC | 2.29 | 1.30 × 10^-12^ |
| ELIM_c3452 | *atpG* | V/A-type Na+-transporting ATPase subunit G/H | 5.33 | 1.53 × 10^-46^ |
| ELIM_c3453 | *atpI* | V/A-type Na+-transporting ATPase subunit I | 4.23 | 2.29 × 10^-45^ |
| ELIM_c3454 | *atpK* | V/A-type Na+-transporting ATPase subunit K | 3.91 | 7.62 × 10^-35^ |
| ELIM_c3455 | *atpE* | V/A-type Na+-transporting ATPase subunit E | 6.03 | 2.34 × 10^-52^ |
| ELIM_c3456 | *atpC* | V/A-type Na+-transporting ATPase subunit C | 5.81 | 1.61 × 10^-69^ |
| ELIM_c3457 | *atpF* | V/A-type Na+-transporting ATPase subunit F | 6.54 | 6.55 × 10^-59^ |
| ELIM_c3458 | *atpA* | V/A-type Na+-transporting ATPase subunit A | 5.50 | 1.37 × 10^-62^ |
| ELIM_c3459 | *atpB* | V/A-type Na+-transporting ATPase subunit B | 4.74 | 6.10 × 10^-51^ |
| ELIM_c3460 | *atpD* | V/A-type Na+-transporting ATPase subunit D | 4.92 | 4.58 × 10^-46^ |
| ELIM_c2347 | *hydC* | NADP-reducing hydrogenase subunit | 1.35 | 1.12 × 10^-5^ |
| ELIM_c2348 | *hydE* | NADP-reducing hydrogenase subunit | 2.07 | 1.18 × 10^-9^ |
| ELIM_c2349 | *hydD* | NADP-reducing hydrogenase subunit | 2.10 | 3.75 × 10^-11^ |
| ELIM_c2350 | *hydB* | NADP-reducing hydrogenase subunit | 2.99 | 2.52 × 10^-23^ |
| ELIM_c2351 | *hydA* | NADP-reducing hydrogenase subunit | 2.27 | 2.74 × 10^-15^ |
| ELIM_c0229 | *fixB* | Electron transfer flavoproteins alpha subunit | 1.48 | 2.08 × 10^-5^ |
| ELIM_c0230 | *fixA* | Electron transfer flavoproteins beta subunit | 0.91 | 1.49 × 10^-2^ |
