# Supplementary material for: Genome-scale analysis of syngas fermenting acetogenic bacteria reveals the translational regulation for its autotrophic growth
Source: BMC Genomics. 2018 Nov 23;19:837. doi: 10.1186/s12864-018-5238-0 (PMC6260860; doi:10.1186/s12864-018-5238-0)
Supplement: Supplementary file 7 — Table S7. Transcription profile of genes associated with central carbon metabolism (DOCX 20 kb) [file 12864_2018_5238_MOESM7_ESM.docx]

**Table S7.** Transcription profile of genes associated with central carbon metabolism

| **Locus Tag** | **Gene** | **Description** | **FC (log2)** | ***P*-value** |
| --- | --- | --- | --- | --- |
| ELIM_c0405 | *glk* | Glucokinase | -0.47 | 4.30 × 10^-1^ |
| ELIM_c2869 | *pgi* | Glucose-6-phosphate isomerase | -0.66 | 7.75 × 10^-2^ |
| ELIM_c2749 | *pfkA* | 6-phosphofructokinase 1 | -1.34 | 1.31 × 10^-4^ |
| ELIM_c0421 | *fbp* | Fructose-1,6-bisphosphatase III | 2.52 | 4.90 × 10^-14^ |
| ELIM_c1783 | *fbaB* | Fructose-bisphosphate aldolase, class I | 3.25 | 4.21 × 10^-24^ |
| ELIM_c1932 | *fbaA* | Fructose-bisphosphate aldolase, class II | 2.37 | 4.03 × 10^-11^ |
| ELIM_c2267 | *fbaA* | Fructose-bisphosphate aldolase, class II | -0.99 | 9.26 × 10^-4^ |
| ELIM_c0107 | *gapA* | Glyceraldehyde 3-phosphate dehydrogenase | -1.59 | 1.49 × 10^-7^ |
| ELIM_c0106 | *pgk* | Phosphoglycerate kinase | -2.04 | 1.68 × 10^-9^ |
| ELIM_c0105 | *tpiA* | Triosephosphate isomerase (TIM) | -1.04 | 1.26 × 10^-3^ |
| ELIM_c3997 | *gpmA* | 2,3-bisphosphoglycerate-dependent phosphoglycerate mutase | 1.83 | 3.10 × 10^-10^ |
| ELIM_c0104 | *gpmI* | 2,3-bisphosphoglycerate-independent phosphoglycerate mutase | -0.56 | 1.23 × 10^-1^ |
| ELIM_c0452 | *eno* | Enolase | -0.25 | 3.78 × 10^-1^ |
| ELIM_c2750 | *pyk* | Pyruvate kinase | -0.92 | 4.06 × 10^-3^ |
| ELIM_c3055 | *ppdK* | Pyruvate phosphate dikinase | 3.68 | 4.16 × 10^-27^ |
| ELIM_c2579 | *pdhA* | Pyruvate dehydrogenase E1 component alpha subunit | -1.75 | 5.24 × 10^-8^ |
| ELIM_c2580 | *pdhA* | Pyruvate dehydrogenase E1 component beta subunit | -0.35 | 2.88 × 10^-1^ |
| ELIM_c2581 | *pdhC* | Pyruvate dehydrogenase E2 component (dihydrolipoamide acetyltransferase) | -0.86 | 6.55 × 10^-3^ |
| ELIM_c2582 | *pdhC* | Dihydrolipoamide dehydrogenase | -0.27 | 4.17 × 10^-1^ |
| ELIM_c3511 | *citA* | Citrate synthase | -0.84 | 7.36 × 10^-3^ |
| ELIM_c1420 | *can* | Aconitate hydratase | -0.26 | 3.81 × 10^-1^ |
| ELIM_c2883 | *idh* | Isocitrate dehydrogenase (NAD+) | -2.70 | 4.51 × 10^-19^ |
| ELIM_c1795 | *korD* | 2-oxoglutarate/2-oxoacid Fd oxidoreductase subunit delta | 5.17 | 4.72 × 10^-28^ |
| ELIM_c1796 | *korA* | 2-oxoglutarate/2-oxoacid Fd oxidoreductase subunit alpha | 4.63 | 3.73 × 10^-30^ |
| ELIM_c1797 | *korB* | 2-oxoglutarate/2-oxoacid Fd oxidoreductase subunit beta | 4.30 | 1.05 × 10^-24^ |
| ELIM_c1798 | *korC* | 2-oxoglutarate/2-oxoacid Fd oxidoreductase subunit gamma | 4.43 | 6.89 × 10^-25^ |
| ELIM_c2301 | *korB* | 2-oxoglutarate/2-oxoacid Fd oxidoreductase subunit beta | 0.01 | 9.84 × 10^-1^ |
| ELIM_c2302 | *korA* | 2-oxoglutarate/2-oxoacid Fd oxidoreductase subunit alpha | 0.33 | 3.41 × 10^-1^ |
| ELIM_c0058 | *pyc* | Pyruvate carboxylase | 0.15 | 6.22 × 10^-1^ |
| ELIM_c3019 | *mdh* | Malate dehydrogenase (oxaloacetate-decarboxylating) | -0.38 | 2.42 × 10^-1^ |
| ELIM_c3042 | *fumA* | Fumarate hydratase subunit alpha | 1.09 | 3.24 × 10^-4^ |
| ELIM_c3043 | *fumB* | Fumarate hydratase subunit beta | 0.57 | 9.72 × 10^-2^ |
| ELIM_c1884 | *pta* | Phosphotransacetylase | 0.84 | 1.01 × 10^-2^ |
| ELIM_c2806 | *ack* | Acetate kinase | 0.14 | 6.61 × 10^-1^ |
| ELIM_c3128 | *aor* | Aldehyde:Fd oxidoreductase | 2.35 | 1.18 × 10^-9^ |
| ELIM_c2953 | *adh* | Alcohol dehydrogenase | 0.10 | 7.55 × 10^-1^ |
| ELIM_c3970 | *adh* | Alcohol dehydrogenase | -1.64 | 2.08 × 10^-7^ |
| ELIM_c2439 | *bdh* | Butanol dehydrogenase | 0.87 | 1.28 × 10^-2^ |
| ELIM_c1634 | *talA* | Transaldolase | -0.15 | 6.78 × 10^-1^ |
| ELIM_c1191 | *tktA* | Transketolase | -0.77 | 9.22 × 10^-3^ |
| ELIM_c1192 | *tktB* | Transketolase | -0.35 | 3.13 × 10^-1^ |
| ELIM_c0631 | *rpe* | Ribulose-phosphate 3-epimerase | -0.58 | 6.58 × 10^-2^ |
| ELIM_c0386 | *rpiB* | Ribose 5-phosphate isomerase B | -1.65 | 1.71 × 10^-7^ |
| ELIM_c1472 | *prsA* | Ribose-phosphate pyrophosphokinase | -0.07 | 8.32 × 10^-1^ |
