# Supplementary figures and images for: Genome-scale analysis of syngas fermenting acetogenic bacteria reveals the translational regulation for its autotrophic growth
Source: BMC Genomics. 2018 Nov 23;19:837. doi: 10.1186/s12864-018-5238-0 (PMC6260860; doi:10.1186/s12864-018-5238-0)

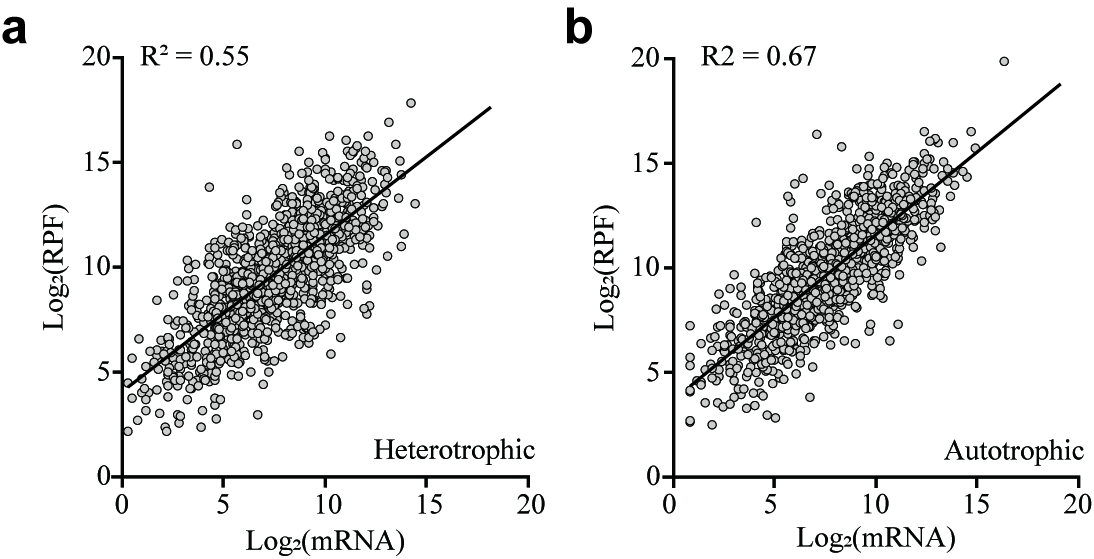

Supplement: Supplementary file 9 — Figure S1. Translatome analysis of E. limosum ATCC 8486. (a) Comparison between RNA-Seq and Ribo-Seq under heterotrophic condition. Scatter plot between ribosome-protected fragment profile (RPF) as the y-axis and mRNA expression (mRNA) as the x-axis. (b) Comparison between RNA-Seq and Ribo-Seq under autotrophic conditions. The results show positive correlation with R2 of 0.55 and 0.67, respectively. (TIF 1104 kb) [file 12864_2018_5238_MOESM9_ESM.tif]

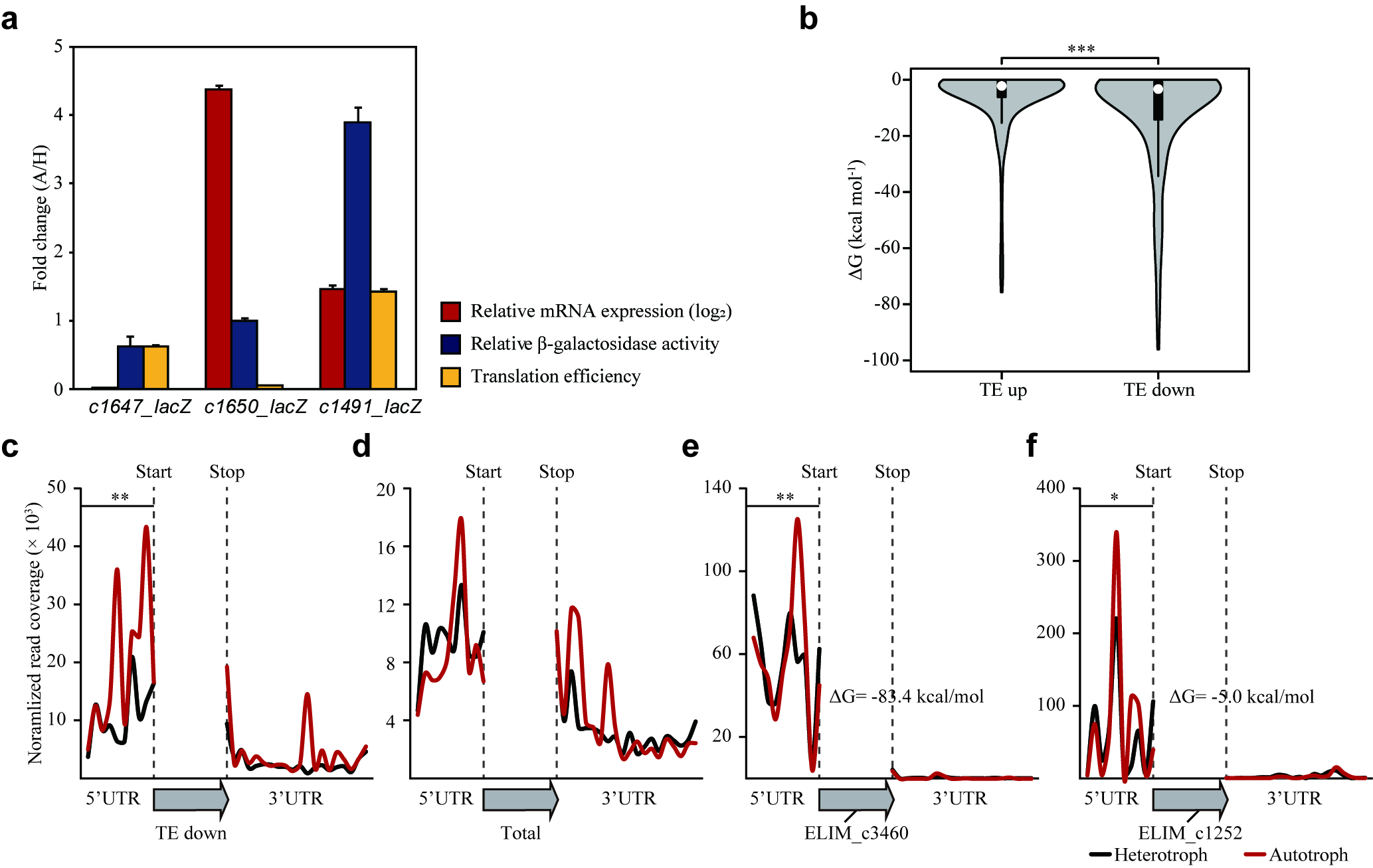

Supplement: Supplementary file 12 — Figure S2. Analysis of translational regulation with determined 5′UTRs. (a) The relative mRNA expression, protein expression, and TE values of ELIM_c1647 (c1647_lacZ), ELIM_c1650 (c1650_lacZ), and ELIM_c1491 (c1491_lacZ) between autotrophic and heterotrophic conditions. TE values were calculated by dividing the relative protein expression obtained from β-galactosidase assay by relative mRNA expression. (b) Box plot showing ΔG (kcal/mol) values of UTRs with median value of − 2.1 and − 3.5 kcal/mol for TE upregulated and downregulated genes, respectively. (c-f) Ribosome coverage across the 5’UTR, coding region, and 3’UTR for decreased TE transcript (c), a total of 1223 genes (d), the ATP synthase cluster transcript (e), and the ribosomal protein biogenesis transcript (f) under the heterotrophic and the autotrophic conditions. The dotted lines indicate translation start and stop sites. The black and red lines represent the ribosome coverage under the heterotrophic and autotrophic conditions, respectively. *P < 0.05; **P < 0.01; ***P < 0.001 (Wilcoxon rank-sum test). (TIF 1563 kb) [file 12864_2018_5238_MOESM12_ESM.tif]
